# Apathy-depression overlap: unmasking hidden disability profiles in multiple sclerosis

**DOI:** 10.3389/fneur.2026.1929740

**Published:** 2026-09-29

**Authors:** Serena Oliveri, Francesca Massaro, Clara Tambussi, Kyrie Piscopo, Matteo Guidetti, Tiziana Zaccone, Alessandra Mingione, Corinne Monzani, Sara Marceglia, Elena Vegni, Alberto Priori, Filippo Martinelli Boneschi

**Affiliations:** 1 Aldo Ravelli Center for Neurotechnology and Experimental Brain Therapeutics, Department of Health Sciences (DISS), University of Milan, Milan, Italy; 2 Clinical Neurology Unit, Azienda Socio Sanitaria Territoriale (ASST) Santi Paolo e Carlo, Milan, Italy; 3 Laboratory of Precision Medicine of Neurological Diseases, Department of Health Sciences, University of Milan, Milan, Italy; 4 Unit of Clinical Psychology, Department of Mental Health and Dependencies, Santi Paolo and Carlo Hospitals, Milan, Italy; 5 Department of Health Sciences, DISS, University of Milan, Milan, Italy

**Keywords:** apathy, depression, disability, fatigue, multiple sclerosis, psychological profile

## Abstract

**Background:**

Multiple sclerosis (MS), the leading cause of non-traumatic neurological disability in young adults, features heterogeneous motor and non-motor symptoms including fatigue, apathy and depression. Although fatigue and depression are well-studied in MS disability progression, to date, the independent and combined contributions of apathy, fatigue and depression to neurological disability remain unexplored.

**Objective:**

This cross-sectional study assessed apathy, depression, and fatigue prevalence in 85 Italian MS patients, their associations with Expanded Disability Status Scale (EDSS) scores, and the relationship between psychological symptom profiles (apathy and/or depression) and disability. Non-parametric analyses and exploratory linear regression models were used.

**Results:**

In our sample of MS patients, the prevalence of apathy, depression and fatigue were 37.6%, 29.3%, and 51.8% respectively. Apathy (r = 0.30, *p* < 0.05) and depression (r = 0.48, *p* < 0.01), but not fatigue (*p* = 0.082), were associated with higher EDSS scores. Patients with co-occurring apathy and depression had significantly higher EDSS than asymptomatic (*p* < 0.01) or apathy-only (*p* < 0.05) groups; isolated apathy or depression were not independently associated with greater disability.

**Discussion:**

Co-occurring apathy and depression may identify an MS subgroup with greater neurological disability at the time of assessment emphasizing the potential value of systematic screening of motivational and affective disturbances in MS by all clinicians, particularly when they co-occur. Our results foster longitudinal studies that might determine whether these psychological profiles may predict future disability progression and whether their identification may inform integrated interventions aimed at improving clinical outcomes.

## Introduction

Multiple sclerosis is a chronic autoimmune disease of the central nervous system, characterized by neuroinflammation, demyelination, and neuroaxonal damage, affecting about 2.8 million people worldwide and is the leading cause of non-traumatic neurological disability, mainly in young adults aged 20–40 and more frequently in women (1–3). Clinical manifestations are highly heterogeneous, reflecting the multifocal distribution of central nervous system lesions and the interplay between inflammatory and neurodegenerative processes (2, 3). Symptoms include motor, cerebellar, sensory, visual, brainstem and sphincter dysfunctions (1, 3), as well as non-motor symptoms such as cognitive impairment, fatigue and psychological changes (4, 5).

Neurological disability represents a core outcome in MS and tends to progress over time, reflecting the cumulative impact of inflammatory and neurodegenerative mechanisms (6). The World Health Organization (WHO) defines disability as arising from the interaction between health condition and contextual factors that limit daily functioning and social participation (7, 8). The Expanded Disability Status Scale (EDSS) serves as the clinical gold standard for quantifying disability in MS (9). Previous studies have shown that male sex, progressive disease course at onset, older age at onset and longer disease duration are among the most consistently reported clinical determinants of disability accumulation in MS and have been strongly associated with higher EDSS scores (6, 10).

Several studies have investigated the role of non-motor neuropsychiatric symptoms in shaping disability outcomes in MS, such as fatigue,^1^ yielding mixed findings, with some reports suggesting a positive association (13, 14) and others supporting the relative independence of fatigue from disability severity (15, 16).

Apathy^2^ and depression are also increasingly recognized as frequent symptoms in people with MS. Depressive symptoms are reported in 20–50% of MS patients with prevalence rates approximately two to three times higher than in the general population (17, 18). Apathy has received considerably less attention in both clinical practice and research (19). Despite this, in recent years, apathy has been recognized as a common symptom in neurodegenerative diseases (20) and MS specifically, with a prevalence estimate ranging from 20% to 45% in MS patients (21, 22). Both conditions have been associated with poorer quality of life, reduced treatment adherence, cognitive deficits, and functional impairment (4, 5). One major challenge is represented by their phenomenological overlap, since fatigue, motivational and psychological disturbances frequently coexist in MS patients and are difficult to disentangle using available assessment tools, which are predominantly based on self-reported rating scales (23–26). Moreover, it remains unclear to what extent these symptoms represent a psychological reaction to chronic illness and/or reflect disease-related neurobiological changes (27, 28). Several cross-sectional and longitudinal studies have reported an association between depressive symptoms and higher EDSS scores, as well as faster disability accrual in MS (7, 29, 30).

However, independent and combined contributions of depressive, motivational and affective disturbances to disability in MS remain poorly understood, limiting our ability to disentangle their respective roles and to characterize distinct clinical profiles associated with greater neurological impairment in MS. The aims of this study were: (1) to assess the prevalence of apathy, depression and fatigue and examine their associations with neurological disability measured by the EDSS in MS patients; (2) to evaluate whether distinct psychological symptoms profiles, defined by the presence of apathy and depression alone or in combination, are differentially associated with disability severity.

Conducting psychological profile-based analysis in MS represents a novel approach: unlike standard assessments focusing on specific symptoms severity alone, profiles capture coping styles, temperament, and emotional responses, offering incremental predictive power for clinical indexes in MS like EDSS scores (31, 32).

## Aims and methods

### Participants

A total of 85 consecutive patients with a diagnosis of RRMS, SPMS, or PPMS, according to McDonald Criteria and further revisions (33) were recruited for this observational cross-sectional study. The study has been conducted at the Multiple Sclerosis Center of San Paolo Hospital, Azienda Socio Sanitaria Territoriale (ASST) Santi Paolo e Carlo, Milan, Italy. Participants were consecutively enrolled during routine outpatient neurological visits between April and July 2025.

Patients with severe psychiatric or cognitive disorders were excluded from the study based on information available in the patients' medical records and on clinical judgement during routine neurological assessment. Patients who were not native Italian speakers were also excluded to ensure adequate comprehension of the clinical questionnaires. All patients provided written informed consent for the retrospective use of their anonymized clinical data for research purposes, in accordance with principles of confidentiality and ethical standards. Ethical approval was obtained from the Milan Area 1 Ethics Committee to which ASST Santi Paolo e Carlo refers, with protocol number 329-2024 dated 06/02/2025.

### Instruments and clinical assessment

All participants underwent a comprehensive clinical evaluation during the neurological visit including:

- detailed medical history;
- data collection from MRI records;
- administration of standardized clinical scales to measure neurological disability, apathy, fatigue, and depressive symptoms.

More in detail, demographic and disease-specific information, including age, sex, age at disease onset, disease duration, MS clinical phenotype, current disease modifying therapies (DMTs), presence of oligoclonal bands (OCBs) in cerebrospinal fluid (CSF), presence of encephalic and spinal cord lesions, use of drugs for the treatment of fatigue such as 4-aminopyridine and depression and serum vitamin D levels and vitamin D supplementation use were collected. Encephalic lesion distribution was further classified according to anatomical location (periventricular, juxtacortical, and infra-tentorial regions). Brain and spinal cord lesion data were obtained from the most recent MRI examination provided by the patients, usually referred to their annual clinical follow-up; presence and lesion location were derived from the corresponding routine neuroradiological reports. The presence of OCBs in cerebrospinal fluid was determined from CSF analysis and was available only for a subgroup of patients.

### Disability assessment

Neurological disability was assessed using the Expanded Disability Status Scale [EDSS; (9)], a clinician-administered scale used during routine neurological examination that classifies MS symptoms according to the degree of disease severity and functional impairment by measuring impairments across different functional systems (pyramidal, cerebellar, brainstem, sensitive, sphincteric, visual, mental) (34). EDSS score ranges from 0 (normal neurological status) to 10 (death due to MS), with higher scores indicating greater disability (35).

### Apathy assessment

Apathy was assessed using the Italian version of the Starkstein's Apathy Scale (SAS) (36, 37), a self-reported questionnaire specifically developed for the evaluation of apathetic symptoms. The SAS consists of 14 items rated on a 4-point Likert scale, yielding a total score ranging from 0 to 42, with higher scores indicating greater apathy severity. In accordance with previous literature, a cut-off score of ≥ 14 was used to identify the presence of clinically significant apathy (37, 38).

### Fatigue assessment

Fatigue severity was evaluated using the Italian version of Fatigue Severity Scale (FSS) (39), a widely used self-reported instrument assessing the impact of fatigue on daily functioning. The FSS is composed of 9 items rated on a 7-point Likert scale (from 1 = “strongly disagree” to 7 = “strongly agree”), with total scores ranging from 9 to 63. Higher scores indicate greater fatigue severity. Consistent with established criteria, a total score > 36 was considered indicative of clinically relevant fatigue (39).

### Depression assessment

Depressive symptoms were assessed using the Patient Health Questionnaire-9 (PHQ-9) (40), a 9-item self-reported questionnaire based on the diagnostic criteria for major depressive disorder. Each item is rated on a 4-point Likert scale (from 0 = “not at all” to 3 = “nearly every day”), resulting in a total score ranging from 0 to 27, with higher scores reflecting greater severity of depressive symptoms. A cut-off score of > 10 was used to identify clinically significant depressive symptomatology (40).

Although apathy, depressive symptoms, and fatigue may partially overlap in MS, the selected tools allow the assessment of distinct clinical dimensions: the SAS specifically targets self-initiated behavior, interest, and emotional concern without confounding physical fatigue with loss of motivation; the PHQ-9 focuses on DSM criteria for major depressive disorder and, although items referring to the “energy” are included, the PHQ-9 maintains strong diagnostic validity for affective disturbance in MS; the FSS was specifically designed to assesses fatigue severity in MS and its impact on daily functioning, rather than motivational drive or mood disorders.

### Statistical analysis and data codification

Statistical analyses were performed using Jamovi (version 2.6.45). Continuous variables were summarized as means and standard deviations, while categorical variables were reported as absolute frequencies and percentages. The normality of data distributions was assessed using the Shapiro-Wilk test. Given the non-normal distribution of variables, non-parametric tests were conducted.

Association between continuous variables were examined using Spearman's rank correlation coefficients. Associations between sex and categorical variables were evaluated using Fisher's exact test. Comparison of EDSS scores between two groups were performed using the Mann-Whitney U test, while comparisons among more than two groups were conducted using the Kruskal-Wallis test.

To examine the association between psychological symptoms and neurological disability, exploratory linear regression models were estimated with EDSS as the dependent variable and the presence of apathy, depressive symptoms, and fatigue, entered as dichotomous variables according to the predefined clinical cut-offs, as predictors. A subsequent model additionally included disease duration as a covariate. Multicollinearity was assessed using the variance inflation factor (VIF).

Psychological symptoms profiles were defined based on dichotomized apathy and depression scores, resulting in four patient groups: (1) asymptomatic profile (Apathy = 0, Depression = 0); (2) pure apathy (Apathy = 1, Depression = 0), (3) pure depression (Apathy = 0, Depression = 1), and (4) co-occurring apathy and depression (Apathy = 1, Depression = 1). No imputation of missing data was performed. Analyses involving depressive symptoms and psychological symptom profiles were conducted using complete cases only.

Differences in EDSS levels across psychological symptoms profiles were analyzed using the Kruskal-Wallis test with *post-hoc* pairwise comparisons performed using the Dwass-Steel-Critchlow-Fligner procedure. Given the exploratory and hypothesis-generating nature of this study, no global correction for multiple testing was applied across the different exploratory analyses, and no *a priori* statistical power analysis was performed.

All statistical tests were two-tailed, and a *p*-value < 0.05 was considered statistically significant.

## Results

The sample consisted of 85 patients diagnosed with multiple sclerosis (mean age: 46.73 years, SD = ±13.83; mean age at disease onset: 33.98 years, SD = ±10.60; mean disease duration: 13 years, SD = ±9.27). The mean EDSS score was 2.43 (SD = ±1.95), the median EDSS score was 2.00 [IQR = 2.50], and mean serum vitamin D levels were 48.45 (SD = ±25.66) ng/mL. Women represented 70.6% of the sample. Relapsing-remitting MS was the most common clinical phenotype (92.9%). Oligoclonal bands (OCB) status in cerebro-spinal fluid (CSF) was available for 56 patients, of whom 42 (75.0%) were OCB-positive and 14 (25.0%) were OCB-negative. Magnetic resonance imaging (MRI) data were available for 74 patients (over 85 included in the assessment). Among them, 100% had brain lesions (75.7% peri-ventricular lesions, 64.9% juxtacortical and 44.6% infra-tentorial) and 75.7% had also spinal cord lesions. Fifty-six patients (65.9% of the total) were treated with lower-efficacy treatment agents (LETA), 25 (29.4% of the total) with high efficacy treatment agents (HETA) and 4 (4.7% of the total) received no disease-modifying drugs (DMD). Treatment with 4-aminopyridine was reported in 20.0% of the sample, while vitamin D supplementation was reported in 92.9% of patients.

Clinically significant apathy was present in 37.6% of patients, fatigue in 51.8% and depressive symptoms in 29.3% of participants. Mean total scores were 12.09 (SD = ±4.64) for apathy, 36.68 (SD = ±14.52) for fatigue and 7.73 (SD = ±5.85) for depressive symptoms, indicating overall mild-to-moderate levels of non-motor symptoms in the sample.

The demographic and clinical aspects of the MS sample are summarized in Table 1.

**Table 1:** Demographic and clinical characteristics of the study sample (*N* = 85 for apathy and fatigue; *N* = 75 for depression; *N* = 74 for MRI data; *N* = 56 for OCB).

Variable	Value *N* (%) or mean ±SD or median [IQR]
**Demographic variables**	
Age, years	46.73 ± 13.83
**Sex**	
Male	25 (29.4)
Female	60 (70.6)
**Clinical variables**	
**MS type**	
RRMS	79 (92.9)
PPMS	4 (4.7)
SPMS	2 (2.4)
EDSS	2.43 ± 1.95, 2.00 [2.50]
Disease duration, years	13.13 ± 9.27
Age at diagnosis, years	33.98 ± 10.60
Vitamin D serum levels, ng/mL	48.45 ± 25.66
**Vitamin D supplementation**	
Yes	79 (92.9)
No	6 (7.1)
**Pharmacological treatment**	
Low efficacy (LETA)	56 (65.9)
High efficacy (HETA)	25 (29.4)
No Treatment	4 (4.7)
**4-aminopyridine**	
Yes	17 (20.0)
No	68 (80.0)
**Oligoclonal bands**	
Yes	42 (75.0)
No	14 (25.0)
**Spinal cord lesion**	
Yes	56 (75.7)
No	18 (24.3)
**Brain lesion**	
Yes	74 (100)
No	0 (0)
**Periventricular lesion**	
Yes	56 (75.7)
No	18 (24.3)
**Juxtacortical lesion**	
Yes	48 (64.9)
No	26 (35.1)
**Infratentorial lesion**	
Yes	33 (44.6)
No	41 (55.4)
**Questionnaires**	
**Apathy presence**	
Yes	32 (37.6)
No	53 (62.4)
SAS	12.09 ± 4.64
**Depression presence**	
Yes	22 (29.3)
No	53 (70.7)
PHQ-9	7.73 ± 5.85
**Fatigue presence**	
Yes	44 (51.8)
No	41 (48.2)
FSS	36.68 ± 14.52

MS, multiple sclerosis; EDSS, Expanded Disability Status Scale; SAS, Starkstein Apathy Scale; PHQ-9, Patient Health Questionnaire-9; FSS, Fatigue Severity Scale.

PHQ-9 data were available for 75 of the 85 participants; missing data reflected assessments that were not collected. Participants with missing PHQ-9 data did not significantly differ from those with available data in age, sex, disease duration, or EDSS (all *p* > 0.05).

As expected, participants' age and disease duration were strongly correlated (r = 0.638, *p* < 0.001). EDSS scores showed a significant positive correlation with both age (r = 0.607, *p* < 0.001) and disease duration (r = 0.341, *p* = 0.001), indicating greater neurological disability with increasing age and, to a lesser extent, longer disease duration.

Among patients with available MRI examination (*n* = 74), no significant associations were found between apathy or fatigue and location of lesion. A weak negative correlation emerged between depressive symptoms and infratentorial lesions (r = −0.271, *p* = 0.028), and between EDSS scores and periventricular lesions (r = −0.241, *p* = 0.039).

Among patients with available CSF data (*n* = 56), no significant associations emerged between apathy, depressive symptoms or fatigue and the presence of oligoclonal bands in CSF.

In the analyzed sample, female sex was associated with a higher prevalence of apathy, fatigue and depressive symptoms. Specifically, apathy was more frequent in women (45%) than men (20%) (OR = 3.27; CI 95%: 1.08–9.87; *p* = 0.048). Fatigue was also more frequent among women (65% vs. 20%) (OR = 7.43; CI 95%: 2.44–22.6; *p* < 0.001). Depressive symptoms were observed exclusively in female participants (39.3% vs. 0%; *p* < 0.001). Also, women showed higher EDSS scores compared with men (Mann–Whitney U = 465, *p* = 0.005; r = 0.38). Results of Mann-Whitney U tests for sex differences are reported in Table 2.

**Table 2:** Sample characteristics stratified by sex.

Variable	Total *N* (%) or mean ±SD and median [IQR]	Female *N* (%) or mean ±SD and median [IQR]	Male *N* (%) or mean ±SD and median [IQR]	*p* value
EDSS	2.4 ± 1.95 2.00 [2.50]	2.78 ± 1.99 2.00 [3.00]	1.60 ± 1.58 1.00 [1.00]	*p* = 0.005^*^
Apathy presence	32/85 (37.6)	27/60 (45.0)	5/25 (20.0)	*p* = 0.048^*^
Depression presence	22/75 (29.3)	22/56 (39.3)	0/19 (0.0)	*p* < 0.001^**^
Fatigue presence	44/85 (51.8)	39/60 (65.0)	5/25 (20.0)	*p* < 0.001^**^

Values are reported as mean ± standard deviation for continuous variables and as percentage (n/N) for categorical variables. EDSS values are reported as mean ± standard deviation and median and interquartile range. Between-group comparisons for EDSS were performed using the Mann–Whitney U test due to non-normal distribution. Associations between sex and categorical variables were assessed using Fisher's exact test. One asterisk indicates statistical significance (*p* < 0.05). Two asterisks indicate statistical significance (*p* < 0.001).

EDSS, Expanded Disability Status Scale.

Bar plots of EDSS scores distribution and prevalence of apathy, depressive symptoms and fatigue stratified by sex are shown below (Figure 1).

**Figure 1:**
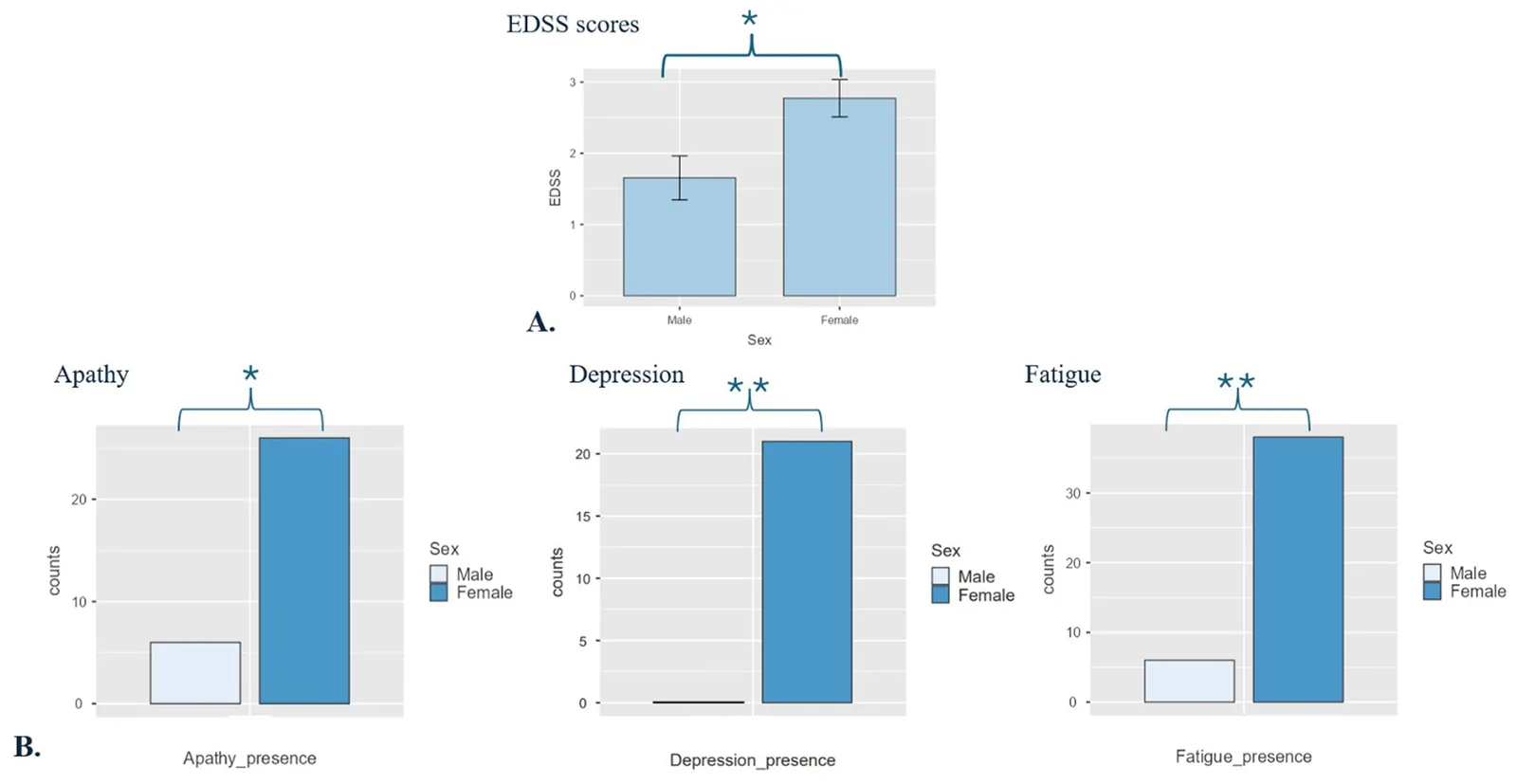
Distribution of EDSS scores and prevalence of apathy, depressive symptoms and fatigue stratified by sex. **(A)** Shows mean EDSS scores by sex [with error bars representing standard deviation (SD)], while **(B)** display the distribution of apathy, depression, and fatigue according to sex. One asterisk indicates statistical significance (*p* < 0.05). Two asterisks indicate statistical significance (*p* < 0.001).

When examining the relationship between non-motor symptoms and neurological disability, our data showed that patients with clinically significant apathy had significantly higher EDSS scores compared to patients without apathy (Mann–Whitney U = 592, *p* = 0.019; r = 0.30). A similar result emerged for depressive symptoms, where patients with depression showed higher neurological disability scores (EDSS scores) (Mann–Whitney U = 305, *p* = 0.001; r = 0.48). In contrast, the presence of clinically significant fatigue did not translate into statistically significant differences in EDSS scores between the two groups of patients (Mann–Whitney U = 706, *p* = 0.082).

Results of Mann-Whitney U tests for EDSS differences are reported in Table 3.

**Table 3:** Comparison of EDSS scores by the presence of apathy, depression and fatigue.

Variable	Group (*N*)	EDSS mean ±SD and median [IQR]	*p* value
Apathy presence	Yes (32)	3.03 ± 2.10, 2.50 [2.50]	*p* = 0.019^*^
	No (53)	2.07 ± 1.77, 1.50 [1.50]	
Depression presence	Yes (22)	3.53 ± 1.92, 3.55 [3.63]	*p* = 0.001^*^
	No (53)	2.01 ± 1.90, 1.50 [1.50]	
Fatigue presence	Yes (44)	2.63 ± 1.77, 2.00 [2.33]	*p* = 0.082
	No (41)	2.22 ± 2.13, 1.50 [2.00]	

EDSS values are reported as mean ± standard deviation and median and interquartile range. EDSS scores were compared between patients with and without each symptom using the Mann–Whitney U test due to non-normal distribution. An asterisk indicates statistical significance (*p* < 0.05). EDSS, Expanded Disability Status Scale.

Boxplots of EDSS distribution according to the presence of apathy, depressive symptoms, and fatigue are shown below (Figure 2).

**Figure 2:**
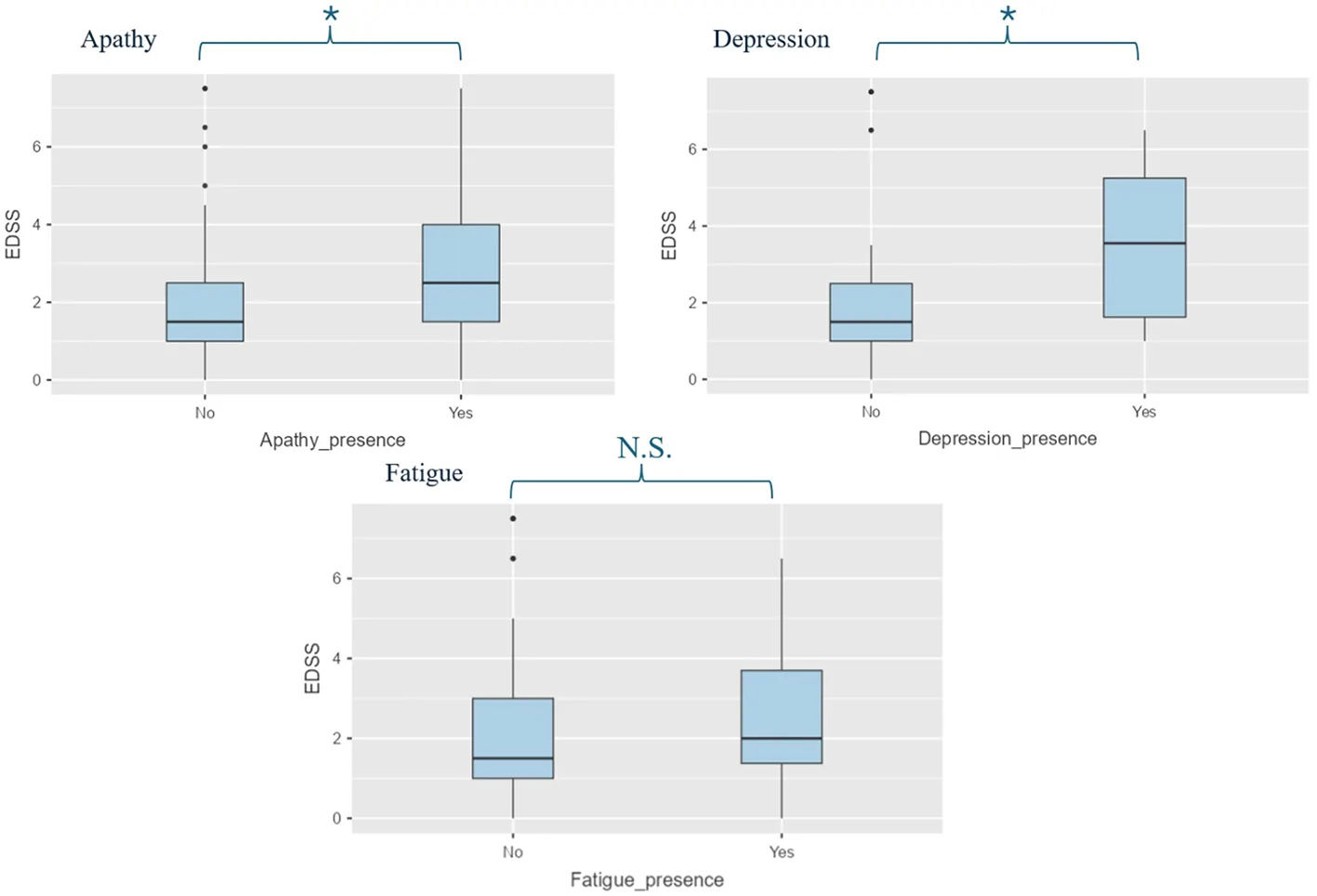
Distribution of EDSS scores according to the presence of apathy, depressive symptoms, and fatigue. Boxes represent the interquartile range (IQR), horizontal lines indicate the median, and whiskers represent the minimum and maximum values; dots represent outliers. An asterisk indicates statistical significance (*p* < 0.05).

To evaluate the independent contribution of apathy, depressive symptoms, and fatigue to neurological disability, an exploratory linear regression analysis was conducted with EDSS score as the dependent variable. In the first model, which included apathy, depressive symptoms and fatigue as predictors, the overall model was statistically significant [F(3,71) = 4.46, *p* = 0.006], explaining 15.9% of the variance in EDSS scores (R^2^ = 0.159; adj R^2^ = 0.123). Within this model, depressive symptoms emerged as the only significant predictor of higher neurological disability (β = 0.82, *p* = 0.006), whereas neither apathy (*p* = 0.168) nor fatigue (*p* = 0.212) showed a significant independent association with EDSS.

Given the clinically relevant role of disease duration as a determinant of neurological disability, a second model was estimated including disease duration as a covariate. The inclusion of disease duration resulted in a significant increase in explained variance (ΔR^2^ = 0.089, *p* = 0.005), yielding a total R^2^ of 0.247 (adj R^2^ = 0.204), and the model remained statistically significant overall [F(4,70) = 5.74, *p* < 0.001]. In this adjusted model, both depressive symptoms (β = 0.82, *p* = 0.004) and disease duration (β = 0.31, *p* = 0.005) were significantly associated with higher EDSS scores, while apathy and fatigue continued to show non-significant independent effects. Variance inflation factor (VIF) values were all below 1.5, indicating the absence of multicollinearity. Full regression coefficients for both exploratory models are reported in Table 4.

**Table 4:** Exploratory linear regression models predicting EDSS.

Predictor	B	95% CI	Standardized β	*p*
**Model 1**				
Apathy presence	0.652	[−0.281, 1.585]	0.324	0.168
Depression presence	1.642	[0.492, 2.793]	0.815	0.006
Fatigue presence	−0.645	[−1.665, 0.376]	−0.320	0.212
**Model 2**				
Apathy presence	0.350	[−0.563, 1.264]	0.174	0.447
Depression presence	1.656	[0.559, 2.752]	0.822	0.004
Fatigue presence	−0.422	[−1.407, 0.564]	−0.105	0.396
Disease duration	0.066	[0.020, 0.113]	0.309	0.005

Model 1: *R*^2^ = 0.159, adjusted *R*^2^ = 0.123, F(3,71) = 4.46, *p* = 0.006. Model 2: *R*^2^ = 0.247, adjusted *R*^2^ = 0.204, F(4,70) = 5.74, *p* < 0.001. B, unstandardized regression coefficient; CI, confidence interval; β, standardized regression coefficient.

We further explored the association between psychological symptoms profiles (asymptomatic profile, pure apathy, pure depression, and co-occurring apathy and depression) and neurological disability (EDSS). The Kruskal-Wallis ANOVA test revealed a significant difference among groups [χ^2^ (3) = 12.8, *p* = 0.005], with an effect size of ε^2^ = 0.176, indicative of a medium-to-large effect.

*Post-hoc* pairwise comparisons using the Dwass–Steel–Critchlow–Fligner procedure showed that the profile characterized by the co-occurrence of apathy and depression (A = 1 and D = 1) exhibited significantly higher EDSS scores compared with the asymptomatic profile (A = 0 and D = 0; W = 4.688, *p* = 0.005) and with the pure apathy profile (A = 1 and D = 0; W = 4.130, *p* = 0.018). No statistically significant differences emerged between other psychological symptom profiles. In the adjusted model, psychological symptom profile remained significantly associated with EDSS after controlling for age, sex, and disease duration (F(3,66) = 6.84, *p* < 0.001). Compared with the asymptomatic profile, the co-occurrence of apathy and depression remained associated with significantly higher EDSS scores (B = 1.37, 95% CI [0.46, 2.28], *p* = 0.004). Age was independently associated with higher EDSS scores (B = 0.10, *p* < 0.001), whereas sex (*p* = 0.167) and disease duration (*p* = 0.484) were not. Description of psychological symptoms profiles and associated EDSS scores are shown in Table 5.

**Table 5:** Summary of psychological symptoms profiles and associated EDSS scores.

Psychological profile	Profile composition	*N* (%)	EDSS mean ±SD and median [IQR]
Asymptomatic	A = 0, D = 0	36 (48.6)	2.03 ± 2.00, 1.50 [1.50]
Pure apathy	A = 1, D = 0	16 (21.6)	1.97 ± 1.76, 1.75 [1.13]
Pure depression	A = 0, D = 1	7 (9.5)	2.64 ± 1.91, 2.00 [2.25]
Apathy + depression	A = 1, D = 1	15 (20.3)	3.94 ± 1.85, 4.00 [3.00]

Profiles were defined based on the presence/absence of apathy and depression. A, Apathy; D, Depression; EDSS; Expanded Disability Status Scale.

Boxplot of EDSS distribution across the four psychological symptoms profiles is shown below (Figure 3).

**Figure 3:**
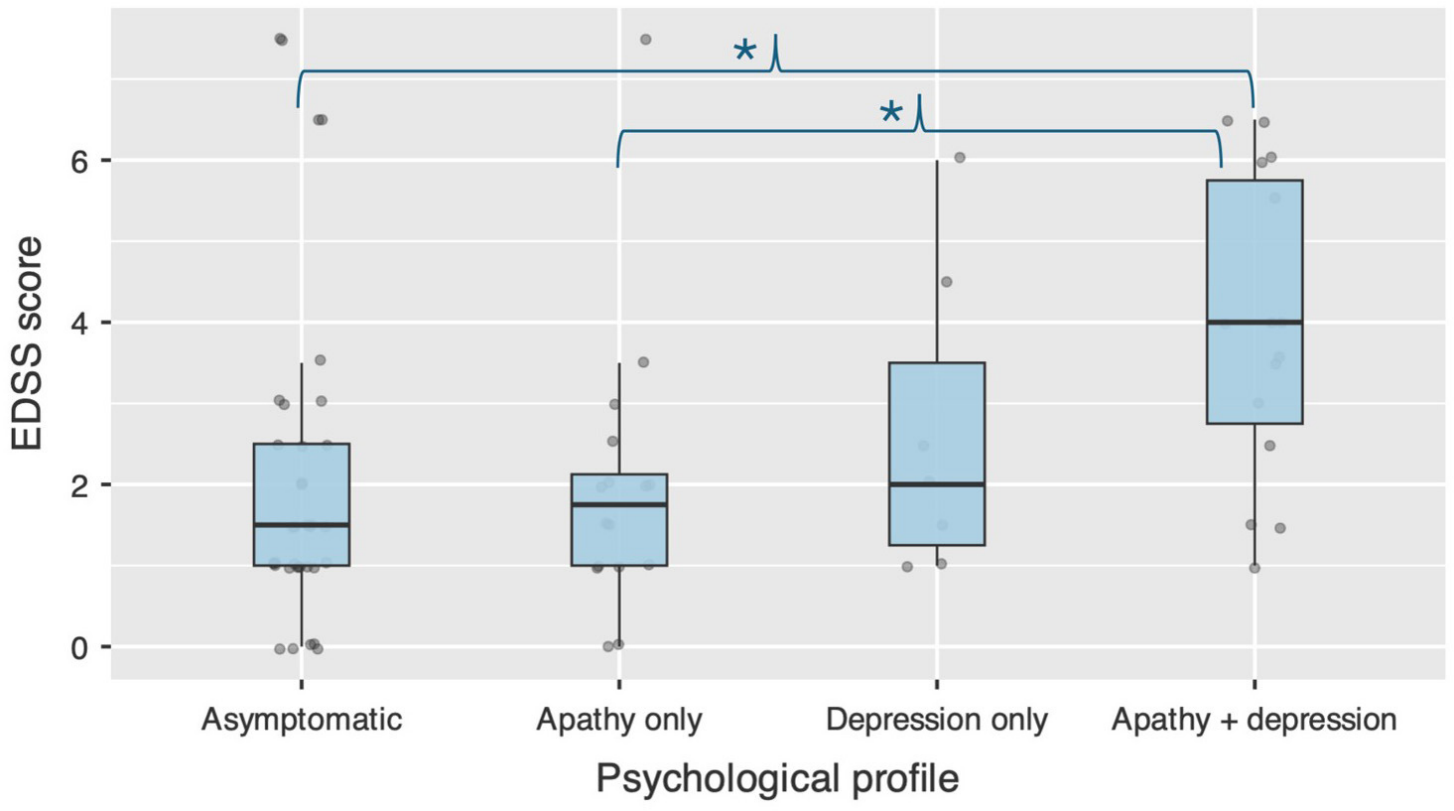
Distribution of EDSS scores across the four psychological symptom profiles. Boxes represent the interquartile range (IQR), horizontal lines indicate the median, whiskers represent the minimum and maximum values, and individual dots represent individual participant observations.

## Discussion

This work contributes to clarifying the complex relationship between apathy, depression and disability in MS. The observed prevalence rates of apathy, depression, and fatigue are broadly consistent with previous literature, confirming that non-motor symptoms are highly prevalent in the MS population (4, 5, 12). Fatigue emerged as the most frequent symptom, affecting over half of the patients of our sample (51.8%), in line with reports indicating fatigue as one of the most common and disabling symptoms in MS (11, 12). Clinically significant apathy was observed in approximately one third of the sample (37.6%), as previously reported (22, 41). Depressive symptoms were present in nearly 30% of evaluated participants, as the well-documented increased burden of depression in MS (18). Even in a cohort of our patients characterized by relatively low-to-moderate levels of neurological disability, these symptoms appear highly prevalent, suggesting that they may occur across the entire spectrum of disease severity. Psychological symptoms were significantly more prevalent among women, with depressive symptoms observed exclusively in female participants, as previously described in MS (13). Nevertheless, our finding should be interpreted with caution since our sample was predominantly composed of females.

Previous studies have also reported associations between lesion characteristics and depression (42) or apathy, finding microstructural damage within frontal cortex, caudate and fronto-striatal white matter bundle as a pathological substrate of apathy (22). In our study we did not find association between apathy or fatigue and lesion distribution instead. These findings should be interpreted cautiously, as MRI data and lesion locations were obtained from routine neurological reports of the most recent available MRI examination (with different intervals between MRI and clinical scales administration across participants) rather than from an MRI screening performed specifically for the purposes of the study. In our further investigations we are planning to adopt more detailed and quantitative MRI measures, such as T2 lesion volume estimation, cortical lesion burden, brain atrophy, or regional volumetric measures to better clarify the neurobiological substrates of psychological symptoms in MS and integrate also cerebrospinal fluid (CSF) data which were now available in a limited number of patients.

When considered independently, apathy and depression, but not fatigue, were associated with higher EDSS scores. Although the lack of association between fatigue and EDSS is also supported by other studies (16), several alternative explanations should be considered. First, the absence of a significant association may reflect the limited statistical power of the present sample. Second, fatigue is a multidimensional construct, that may be shaped by psychological factors beyond mere physical disability. Third, the fatigue scale used [FSS; (39)] primarily captured the severity and functional impact of fatigue but does not distinguish between its different physical, cognitive, and psychosocial dimensions. Furthermore, the relatively restricted range of EDSS scores in our sample may have limited the ability to detect an association between fatigue and neurological disability.

In contrast, in our study depressive symptoms remained an independent predictor of EDSS severity also after controlling for disease duration, as confirmed in other contributions (7, 30).

Although apathy was associated with higher EDSS in univariable analyses, it did not emerge as an independent predictor of disability in adjusted statistical models. Apathy often co-occurs with depression, fatigue and cognitive deficits in MS; multivariable adjustments likely attenuate its effect by accounting for these shared pathways. Thus, to better clarify the role of apathy in MS disability, we conducted psychological profile-based analysis defined by the co-occurrence of apathy and depression. These analysis suggested that apathy may be clinically relevant primarily when coexisting with depression, as patients presenting with both apathy and depressive symptoms had greater neurological disability at the time of assessment compared with the other psychological symptoms profiles. This cluster of patients must be identified early to manage key factors determining their daily functioning. However, longitudinal studies are needed to determine whether depression, in co-occurrence with apathy, might be specifically associated with future disability progression and to investigate if targeted interventions may improve clinical outcomes.

Overall, the profile-based assessment of non-motor neuropsychiatric symptoms, and the investigation of potential targeted intervention, are particularly relevant given that MS predominantly affects young adults during their most productive, economically and socially active phases of their life, amplifying the long-term implications of psychological comorbidities in terms of quality of life, functional outcomes and cognitive complaints (4, 5).

Systematic and multidimensional screening of apathy and depression in all MS patients is not only a responsibility of neurologists, but it also involves the collaboration of psychologists, physiotherapists and primary care physicians who manage the long-term monitoring of the disease. Such screening may facilitate the early identification of patients with greater neurological disability and support a more comprehensive assessment of their clinical and psychological needs.

We should recognize that the small and unbalanced size of the four psychological profile subgroups identified in our study (particularly the pure depression subgroup) may have limited the statistical power to detect differences across profiles or observe significant variance explanation in EDSS; therefore, non-significant findings observed for pure apathy and depression should be interpreted cautiously.

Indeed we are currently conducting longitudinal follow-up assessments at 6, 12, and 24 months in the study cohort and enlarging the enrollment, including repeated administration of the clinical scales, to investigate changes in EDSS and transitions between psychological symptoms profiles over time. Since most participants had a RRMS phenotype of disease and a relatively low EDSS score, caution is needed in generalizing results to a wider and more heterogeneous MS sample, and larger multicenter studies are required to replicate and extend these findings and increase the sample of other MS disease courses like SPMS and PPMS and MS patients with increasing disability.

## Data Availability

The raw data supporting the conclusions of this article will be made available by the authors, without undue reservation.

## Footnotes

1 Fatigue is recognized as one of the most prevalent and disabling non-motor symptoms in multiple sclerosis, affecting 70-80% of MS patients and represents one of main causes of impaired quality of life. It is a multidimensional symptom defined as a lack of physical or mental energy, affecting social, cognitive, and daily functioning domains (11, 12).

2 Apathy is a multidimensional neurobehavioral syndrome defined as a lack of motivation, initiative and emotional responsiveness not attributable to diminished level of consciousness, cognitive decline or emotional distress (43).
